# Total bilirubin level is associated with acute kidney injury in neonates admitted to the neonatal intensive care units: based on MIMIC-III database

**DOI:** 10.1007/s00431-024-05682-5

**Published:** 2024-07-11

**Authors:** Huan Zhou

**Affiliations:** Department of Neonatology, The Central Hospital of Wuhan, Tongji Medical College, Huazhong University of Science and Technology, No.26 Shengli Street, Jiangan District, Wuhan, 430014 Hubei Province China

**Keywords:** Neonates, Acute kidney injury, Total bilirubin, Protective factor, Neonatal intensive care units

## Abstract

**Objective:**

The objective of this study was to investigate the association between total bilirubin and acute kidney injury (AKI) in neonates admitted to neonatal intensive care units (NICU).

**Methods:**

All data utilized were extracted from Medical Information Mart for Intensive Care-III (MIMIC-III) in this retrospective cohort study. The primary outcome was the occurrence of AKI during hospitalization in the NICU, and the exposure was the initial measurement of total bilirubin levels within 24 h of neonatal admission to the NICU. The relationship between serum total bilirubin and AKI was evaluated by employing univariate and multivariate logistic regression models. Additionally, subgroup analyses were conducted based on birth weight, sepsis, and mechanical ventilation.

**Results:**

This retrospective cohort study included a population of 1,726 neonates, and 95 neonates developed AKI. Total bilirubin, as a continuous variable, was linked with decreased AKI risk among neonates admitted to the NICU [odds ratio (OR) = 0.77, 95% confidence interval (CI): 0.64–0.92]. Similarly, when total bilirubin levels were categorized by tertiles, tertiles 3 showed a significant association with decreased AKI risk (OR = 0.39, 95%CI: 0.19–0.83). The relationship of total bilirubin level and AKI was also existent among neonates admitted to the NICU who were underweight, had not sepsis, and received mechanical ventilation.

***Conclusion*:**

Total bilirubin level may be a protective factor for the risk of developing AKI.

**Supplementary information:**

The online version contains supplementary material available at 10.1007/s00431-024-05682-5.

## Introduction

Acute kidney injury (AKI) is regarded as a complication in newborns admitted to the neonatal intensive care units (NICU) [1]. It is typically characterized by a sudden decline in renal function, leading to disturbances in fluid and electrolyte balance, alteration of acid–base homeostasis, as well as accumulation of waste products [2, 3]. According to reports, neonatal AKI has been linked to various adverse outcomes, such as increased mortality risk, prolonged hospitalization periods, and escalated healthcare expenditures [4, 5]. Therefore, timely identification of neonatal AKI risk is of great significance.

Bilirubin, serving as a reliable indicator of liver function, is the final product resulting from the breakdown of heme within blood vessels [6, 7]. It has been demonstrated that bilirubin possesses potent antioxidant properties, enabling it to potentially reverse or prevent damage caused by reactive oxygen species (ROS) produced during ischemia and reperfusion [8]. There is some evidence to suggest that elevated levels of bilirubin may confer a protective effect against certain types of chronic kidney diseases. For example, in the study of Aoki Y et al., they found that a correlation exists between lower serum bilirubin levels and a more pronounced decline in kidney function [9]. A study has suggested that decreased levels of indirect bilirubin may be correlated with an elevated risk of sepsis-induced AKI [10]. In addition, total bilirubin also exhibited a protective effect on neonatal mortality within the NICU [11]. Nevertheless, based on our current understanding, the investigation into the association between bilirubin and the risk of AKI in newborns within NICU has not been sufficiently explored.

The current study aimed to explore the correlation between bilirubin and AKI risk of neonates in the NICU, utilizing data from the Medical Information Mart for Intensive Care III (MIMIC-III) database, which provided further evidence for AKI prevention strategies in NICU.

## Methods

### Data sources

The information for this research was obtained from the MIMIC-III database. MIMIC-III is a comprehensive and openly accessible database that encompasses de-identified health data for over 40,000 intensive care unit (ICU) patients treated at the Beth Israel Deaconess Medical Center between 2001 and 2012 [12], including demographics, vital signs, medications and laboratory tests, imaging reports, duration of hospital stay, and survival outcomes [13]. The data included in this study were downloaded from a public database, and all patient information was de-identified (https://physionet.org/content/mimiciii/1.4/). Thus, there was no need of ethic approval from the ethics committee at Central Hospital of Wuhan, Tongji Medical College, Huazhong University of Science and Technology.

### Participants

**Fig. 1:**
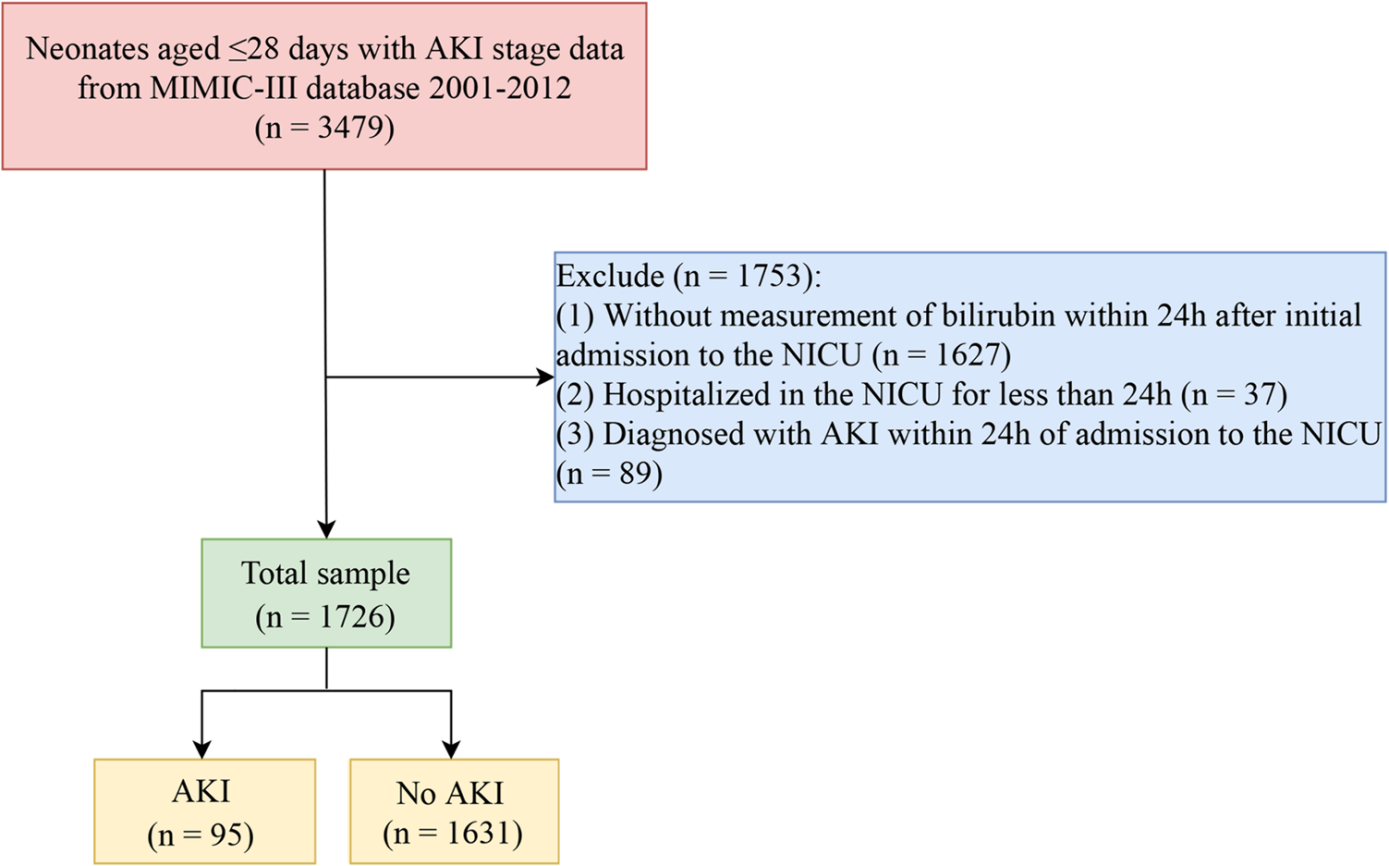
Overview of study subjects’ selection

Neonates aged ≤ 28 days with AKI stage data from MIMIC-III database 2001–2012 were selected for this retrospective cohort study. Exclusion criteria: (1) without measurement of bilirubin within 24 h after initial admission to the NICU; (2) the length of stay in NICU was less than 24 h; (3) diagnosed with AKI within 24 h of admission to the NICU. The selection process for study participants is depicted in Fig. 1. Eventually, 1,726 newborns were included in the retrospective cohort study.

### Study endpoint

The study outcome of this study was AKI occurrence in NICU patients during their hospitalization. The neonatal AKI was defined based on the criteria established by Kidney Disease Improving Global Outcomes (KDIGO) criteria: a rise in serum creatinine (SCr) of ≥ 0.3 mg/dL within 48 h, or an increase of ≥ 1.5 times from the initial level, or a urine output < 0.5 ml/kg/h for a duration of at least 6 h [4]. The initial ICU admission of the patient was designated as the start date of follow-up, with a median follow-up time of 16.57 (7.44, 39.65) days.

### Exposure

The exposure of this study was the initial measurement of total bilirubin levels within 24 h of neonatal admission to the NICU. The total bilirubin levels were classified into three categories based on the tertiles, tertiles 1: < 4.5 mg/dL, tertiles 2: 4.5–5.7 mg/dL, and tertiles 3: ≥ 5.7 mg/dL.

### Clinic variables

The following variables were obtained: age (years), ethnicity, gender, birth weight (kg), renal agenesis and dysgenesis, urinary tract infection, sepsis, respiratory distress syndrome, asphyxia, patent ductus arteriosus, necrotizing enterocolitis, heart rate (bpm), respiratory rate (bpm), bicarbonate (mEq/L), potassium (mEq/L), sodium (mEq/L), chloride (mEq/L), urine output (mL), vasopressors, mechanical ventilation, vancomycin, nonsteroidal anti-inflammatory drug, angiotensin-converting enzyme (ACE) inhibitors, amphotericin B, acyclovir or valacyclovir, and length of stay (Los, days). The collection of vital signs and laboratory data occurred within 24 h following admission to the NICU.

### Statistical analysis

In this study, variables were removed if the percentage of missing values exceeded 20%. For variables with missing values below 20%, random forest interpolation was applied to fill in. A comparative analysis was conducted using the data before and after imputation (Supplemental Table 1). Measured data with normal distribution was described using Mean ± standard deviation (Mean ± SD), while non-normal distribution was described using median and quartiles [M (Q1, Q3)]. The groups were compared for differences using t-test and Mann–Whitney U test, respectively. The categorical data were represented as the number of cases and the constituent ratio [N (%)], and group comparisons were conducted using χ^2^ test. *P* < 0.050 was considered statistically significant.

We utilized univariate logistic regression analysis to explore confounding factors that may influence the risk of AKI in neonates with NICU. Then, we employed both univariate and multivariate logistic regression models to investigate the association between total bilirubin levels and risk of AKI during hospitalization in the NICU. Odd ratio (OR) with 95% confidence interval (CI) were calculated. We calculated the variance inflation factor (VIF) to assess the collinearity among the selected covariates (collinearity was considered when VIF > 10.0). All statistical analyses were performed using Python 3.9.12, SAS 9.4 (SAS Institute Inc., Cary, NC, USA) and R 4.2.2.

## Results

### Baseline characteristics

The baseline characteristics of all eligible neonates were summarized in Table 1 (n = 1,726). The median age of all included neonates was 0.59 (0.35, 0.83) years, and 54.4% were male. 95 neonates developed AKI and 1,631 neonates did not develop AKI. The median total bilirubin level in neonates diagnosed with AKI was found to be significantly lower compared to those without AKI (3.70 mg/dL *vs* 5.20 mg/dL). AKI group exhibited significantly elevated heart rate (157.40 ± 16.80 bpm *vs* 152.23 ± 15.97 bpm), chloride level (108.01 ± 5.64 mEq/L *vs* 106.13 ± 4.33 mEq/L), and urine output [1256.17 (601.00, 2206.00) mL *vs* 582.00 (292.00, 957.00) mL] compared to the non-AKI group. In addition, there were notable disparities in certain baseline characteristics between AKI group and non-AKI group, including birth weight, sepsis, respiratory distress syndrome, mechanical ventilation, patent ductus arteriosus, vasopressor, nonsteroidal anti-inflammatory drug, and acyclovir or valacyclovir (*P* < 0.050).

**Table 1:** Baseline characteristics of all eligible neonates

Variables	Total (n = 1726)	Groups		*P*
		No-AKI group (n = 1631)	AKI group (n = 95)	
Age, years, M (Q_1_, Q_3_)	0.59 (0.35, 0.83)	0.59 (0.35, 0.83)	0.56 (0.35, 0.80)	0.385
Gender, n (%)				0.482
Female	787 (45.60)	747 (45.80)	40 (42.11)	
Male	939 (54.40)	884 (54.20)	55 (57.89)	
Ethnicity, n (%)				0.628
Black	212 (12.28)	200 (12.26)	12 (12.63)	
Others	304 (17.61)	291 (17.84)	13 (13.68)	
Unknown	153 (8.86)	142 (8.71)	11 (11.58)	
White	1057 (61.24)	998 (61.19)	59 (62.11)	
Birth weight, kg, M (Q_1_, Q_3_)	1.79 (1.28, 2.31)	1.82 (1.34, 2.32)	0.96 (0.72, 1.68)	< 0.001
Birth weight, n (%)				0.003
Normal/Overweight	326 (18.89)	319 (19.56)	7 (7.37)	
Underweight	1400 (81.11)	1312 (80.44)	88 (92.63)	
Renal agenesis and dysgenesis, n (%)				1.000
No	1724 (99.88)	1629 (99.88)	95 (100.00)	
Yes	2 (0.12)	2 (0.12)	0 (0.00)	
Urinary tract infection, n (%)				1.000
No	1722 (99.77)	1627 (99.75)	95 (100.00)	
Yes	4 (0.23)	4 (0.25)	0 (0.00)	
Sepsis, n (%)				0.002
No	1641 (95.08)	1558 (95.52)	83 (87.37)	
Yes	85 (4.92)	73 (4.48)	12 (12.63)	
Respiratory distress syndrome, n (%)				< 0.001
No	840 (48.67)	813 (49.85)	27 (28.42)	
Yes	886 (51.33)	818 (50.15)	68 (71.58)	
Asphyxia, n (%)				0.494
No	1718 (99.54)	1623 (99.51)	95 (100.00)	
Yes	8 (0.46)	8 (0.49)	0 (0.00)	
Patent ductus arteriosus, n (%)				< 0.001
No	1460 (84.59)	1416 (86.82)	44 (46.32)	
Yes	266 (15.41)	215 (13.18)	51 (53.68)	
Necrotizing enterocolitis, n (%)				0.281
No	1685 (97.62)	1594 (97.73)	91 (95.79)	
Yes	41 (2.38)	37 (2.27)	4 (4.21)	
Length of stay in NICU, days, M (Q_1_, Q_3_)	18.21 (8.36, 43.19)	17.35 (8.22, 41.24)	53.30 (17.73, 96.43)	< 0.001
Heart rate, bpm, Mean ± SD	152.23 ± 15.97	151.93 ± 15.88	157.40 ± 16.80	0.001
Respiratory rate, bpm, Mean ± SD	48.44 ± 10.60	48.51 ± 10.56	47.28 ± 11.31	0.273
Bicarbonate, mEq/L, Mean ± SD	21.23 ± 2.70	21.24 ± 2.67	20.99 ± 3.25	0.451
Sodium, mEq/L, Mean ± SD	138.97 ± 4.30	138.93 ± 4.24	139.77 ± 5.23	0.127
Potassium, mEq/L, Mean ± SD	4.99 ± 0.99	5.00 ± 0.99	4.83 ± 0.97	0.100
Chloride, mEq/L, Mean ± SD	106.23 ± 4.44	106.13 ± 4.33	108.01 ± 5.64	0.002
Urine output, mL, M (Q_1_, Q_3_)	595.00 (306.00, 1015.00)	582.00 (292.00, 957.00)	1256.17 (601.00, 2206.00)	< 0.001
Mechanical ventilation, n (%)				< 0.001
No	553 (32.04)	542 (33.23)	11 (11.58)	
Yes	1173 (67.96)	1089 (66.77)	84 (88.42)	
Vasopressors, n (%)				< 0.001
No	1555 (90.09)	1495 (91.66)	60 (63.16)	
Yes	171 (9.91)	136 (8.34)	35 (36.84)	
Vancomycin, n (%)				< 0.001
No	1494 (86.56)	1435 (87.98)	59 (62.11)	
Yes	232 (13.44)	196 (12.02)	36 (37.89)	
ACE inhibitor, n (%)				0.156
No	1723 (99.83)	1629 (99.88)	94 (98.95)	
Yes	3 (0.17)	2 (0.12)	1 (1.05)	
Nonsteroidal anti-inflammatory drug, n (%)				< 0.001
No	1365 (79.08)	1327 (81.36)	38 (40.00)	
Yes	361 (20.92)	304 (18.64)	57 (60.00)	
Amphotericin B, n (%)				0.554
No	1720 (99.65)	1625 (99.63)	95 (100.00)	
Yes	6 (0.35)	6 (0.37)	0 (0.00)	
Acyclovir or valacyclovir, n (%)				0.002
No	1708 (98.96)	1618 (99.20)	90 (94.74)	
Yes	18 (1.04)	13 (0.80)	5 (5.26)	
Follow time, days, M (Q_1_, Q_3_)	16.57 (7.44, 39.65)	17.35 (8.22, 41.24)	5.25 (4.17, 13.54)	< 0.001
Total bilirubin, mg/dL, M (Q_1_, Q_3_)	5.20 (4.10, 6.10)	5.20 (4.20, 6.20)	3.70 (2.70, 4.90)	< 0.001
Total bilirubin, n (%)				< 0.001
Tertiles 1	554 (32.10)	489 (29.98)	65 (68.42)	
Tertiles 2	554 (32.10)	535 (32.80)	19 (20.00)	
Tertiles 3	618 (35.81)	607 (37.22)	11 (11.58)	

*AKI* = acute kidney injury; *NICU* = neonatal intensive care units; *ACE* = angiotensin-converting enzyme

### Association between total bilirubin and AKI

As shown in Supplemental Table 2, the result of univariate logistic regression analysis presented that birth weight, sepsis, respiratory distress syndrome, patent ductus arteriosus, heart rate, chloride, urine output, vasopressors, mechanical ventilation, vancomycin, nonsteroidal anti-inflammatory drug, and acyclovir or valacyclovir were confounding factors in this study (*P* < 0.050) [14–18]. Supplemental Table 3 also revealed that the VIF values for all selected covariates were all below 10, indicating the absence of collinearity in our study. In addition, we used the Hosmer–Lemeshow goodness-of-fit to test the goodness-of-fit. The *P* value (*P* = 0.398) is greater than 0.05, it indicates that the model has a good fit.

Subsequently, we employed univariate and multivariate logistic regression models to determine the correlation of total bilirubin levels with risk of AKI during hospitalization in the NICU (Table 2). In the univariate logistic regression model, it was observed that total bilirubin, when considered as a continuous variable, were linked to a reduction in the risk of AKI among neonates admitted to the NICU (OR = 0.54, 95%CI: 0.47–0.63, *P* < 0.001). After controlling for all confounding factors, a statistically significant correlation persists between levels of total bilirubin and the likelihood of AKI (OR = 0.77, 95%CI: 0.64–0.92, *P* = 0.004). Similarly, when total bilirubin levels were categorized by tertiles, with tertiles 1 as the reference group, both tertiles 2 and 3 exhibited a significant correlation with a reduced risk of AKI in the univariate logistic regression model. However, the multivariate logistic regression model revealed that tertiles 3 of total bilirubin level was related to a decreased risk of AKI for neonates admitted to the NICU (OR = 0.39, 95%CI: 0.19–0.83). Tests for trends were statistically significant (*P* = 0.011).

**Table 2:** Association between total bilirubin and AKI

Variables	Univariate model		Multivariate model	
	OR (95%CI)	*P*	OR (95%CI)	*P*
Total bilirubin	0.54 (0.47–0.63)	< 0.001	0.77 (0.64–0.92)	0.004
Total bilirubin				
Tertiles 1	Ref		Ref	
Tertiles 2	0.27 (0.16–0.45)	< 0.001	0.65 (0.35–1.18)	0.156
Tertiles 3	0.14 (0.07–0.26)	< 0.001	0.39 (0.19–0.83)	0.014
*P for trend testing*		< 0.001		0.011

*AKI* = acute kidney injury; *OR* = odd ratio; *CI* = confidence interval

Univariate model: did not adjust any variables

Multivariate model: adjusted for birth weight, sepsis, respiratory distress syndrome, patent ductus arteriosus, heart rate, chloride, urine output, vasopressors, mechanical ventilation, vancomycin, nonsteroidal anti-inflammatory drug, and acyclovir or valacyclovir

## Discussion

This study observed that neonates admitted to the NICU who had higher levels of total bilirubin were less likely to develop AKI in the fully adjusted model. Therefore, the measurement of total bilirubin may potentially serve as a valuable biomarker for predicting the likelihood of AKI occurrence, thus providing novel evidence to inform strategies for preventing AKI in NICU settings.

The incidence of AKI has been reported to be higher in newborns compared to many other populations with critical kidney diseases [19]. A study pointed out that newborns may face an increased risk of developing AKI during the initial days after birth due to some characteristic, such as elevated renal vascular resistance and plasma renin activity, as well as reduced glomerular filtration rate (GFR) and intercortical perfusion [1]. In this study, the prevalence of AKI in neonates approximately was 5.5%. In addition, we observed a significant decrease in the median total bilirubin level among neonates diagnosed with AKI compared to those without AKI, suggesting an inverse correlation between AKI and total bilirubin levels. It is widely acknowledged that the presence of antioxidant and anti-inflammatory properties [20] in bilirubin could potentially be linked to an increased susceptibility to certain diseases when total bilirubin levels are lower [9, 21]. Some available evidence also suggests a potential association between bilirubin and AKI. In a systematic evaluation and meta-analysis conducted by Lyu L et al., total bilirubin levels were found to have a positive correlation with the contrast-induced acute kidney injury (CI-AKI) occurrence; furthermore, both low and high concentrations of bilirubin were found to be associated with an increased risk of CI-AKI, with a higher incidence observed in the group with low bilirubin concentrations compared to those with higher bilirubin concentrations [22]. However, there is still uncertainty about the association between neonatal bilirubin and AKI. Compared with previous studies on AKI, the present study has new finding. Total bilirubin levels may act as a potential protective factor against the risk of AKI among neonates admitted to the NICU after adjusting for birth weight, sepsis, respiratory distress syndrome, patent ductus arteriosus, heart rate, chloride, urine output, vasopressors, mechanical ventilation, vancomycin, nonsteroidal anti-inflammatory drug, and acyclovir or valacyclovir, indicating an association of bilirubin levels with a reduced likelihood of developing AKI.

The underlying mechanisms responsible for the protective effects of total bilirubin on AKI in neonates admitted to the NICU remain elusive. The bilirubin is considered as a byproduct of heme catabolism [23], and it can also serve as a measure for assessing liver function. Existing evidence strongly supports the notion that bilirubin, as very effective physiological antioxidant, may possess a greater capacity to suppress oxidative stress compared to other antioxidant agents [24], and exert inhibitory effects on lipid and lipoprotein oxidation under physiological conditions [25, 26]. In addition, multiple research investigations have suggested the pivotal role of oxidative stress in the pathogenesis of AKI [27, 28]. Consequently, an elevation in bilirubin levels may result in heightened antioxidant activity, thereby mitigating the risk of AKI. It should be noted that when neonatal bilirubin levels are high, the clinical manifestation is icterus [29]. Phototherapy is the most prevalent, most effective, and least dangerous treatment method for neonatal hyperbilirubinemia, representing the primary treatment option for neonatal icterus [30]. Additionally, phototherapy has been shown to potentially enhance urinary calcium excretion [30]. Receiving phototherapy in newborns may lead to an elevation in urinary nitrogen monoxide production, potentially resulting in hemodynamic changes [31]. Newborns with low gestational age or birth weight are particularly susceptible to AKI. When they exhibit elevated bilirubin levels, phototherapy may enhance neurodevelopmental outcomes but could potentially elevate mortality rates, especially among the smallest and sickest infants [32, 33].

Unfortunately, our study is subject to several limitations as follows. Firstly, the data utilized in this research were solely acquired from single-center, and the generalizability of our findings to the broader population remains uncertain. Different genetic background was linked with jaundice natural trend, and transcutaneous bilirubin levels had some differences across populations [34]. Secondly, the MIMIC-III database was deficient in maternal-related information, such as maternal age, body mass index, intrapartum related complication, and gestational age, which may be covariates in this study [35, 36]. Lastly, the retrospective study design of research limits the power of our results. Future investigations should be conducted as a multicenter study, encompassing a large sample of cases, and undertaking further analysis and validation of the findings from this study.

## Conclusion

In summary, our findings indicated an association of total bilirubin with reduced risk of AKI among neonates admitted to the NICU. The measurement of total bilirubin may have the potential to be utilized as a valuable biomarker for predicting likelihood of developing AKI, thereby offering novel evidence to guide preventive strategies in NICU settings.

## Supplementary information

Below is the link to the electronic supplementary material.

Supplementary file1 (DOCX 16 KB)

Supplementary file2 (DOCX 18 KB)

Supplementary file3 (DOCX 15 KB)

## Data Availability

The datasets used and/or analysed during the current study available from the corresponding author on reasonable request.
