# Supplementary material for: Total bilirubin level is associated with acute kidney injury in neonates admitted to the neonatal intensive care units: based on MIMIC-III database
Source: Eur J Pediatr. 2024 Jul 11;183(10):4235–41. doi: 10.1007/s00431-024-05682-5 (PMC11413182; doi:10.1007/s00431-024-05682-5)
Supplement: Supplementary file 1 — Supplementary file1 (DOCX 16 KB) [file 431_2024_5682_MOESM1_ESM.docx]

**Supplemental Table 1 Sensitivity analysis of before and after interpolation**

| **Variables** | **Before interpolation** | **After interpolation** | **Statistics** | ***P*** |
| --- | --- | --- | --- | --- |
| Potassium, Mean ± SD | 4.98 ± 1.02 | 4.99 ± 0.99 | t=0.29 | 0.772 |
| Urine output, M (Q_1_, Q_3_) | 593.00 (307.00, 1015.00) | 595.00 (306.00, 1015.00) | Z=0.008 | 0.993 |
| Respiratory rate, Mean ± SD | 48.42 ± 11.47 | 48.44 ± 10.60 | t=0.05 | 0.960 |
| Sodium, Mean ± SD | 138.98 ± 4.46 | 138.97 ± 4.30 | t=-0.05 | 0.964 |
| Chloride, Mean ± SD | 106.26 ± 4.59 | 106.23 ± 4.44 | t=-0.17 | 0.861 |
| Bicarbonate, Mean ± SD | 21.22 ± 2.82 | 21.23 ± 2.70 | t=0.13 | 0.897 |
| Birth weight, M (Q_1_, Q_3_) | 1.79 (1.27, 2.31) | 1.79 (1.28, 2.31) | Z=-0.043 | 0.966 |
| Heart rate, Mean ± SD | 152.15 ± 16.05 | 152.23 ± 15.97 | t=0.15 | 0.884 |
