# Supplementary material for: Total bilirubin level is associated with acute kidney injury in neonates admitted to the neonatal intensive care units: based on MIMIC-III database
Source: Eur J Pediatr. 2024 Jul 11;183(10):4235–41. doi: 10.1007/s00431-024-05682-5 (PMC11413182; doi:10.1007/s00431-024-05682-5)
Supplement: Supplementary file 2 — Supplementary file2 (DOCX 18 KB) [file 431_2024_5682_MOESM2_ESM.docx]

**Supplemental Table 2 Screening of confounding factors related to AKI by univariate logistic regression analysis**

| Variables | OR (95%CI) | *P* |
| --- | --- | --- |
| Age | 0.64 (0.39-1.05) | 0.076 |
| Gender |  |  |
| Female | Ref |  |
| Male | 1.16 (0.76-1.77) | 0.482 |
| Ethnicity |  |  |
| Black | Ref |  |
| Others | 0.74 (0.33-1.67) | 0.473 |
| Unknown | 1.29 (0.55-3.01) | 0.554 |
| White | 0.99 (0.52-1.87) | 0.964 |
| Birth weight | 0.23 (0.16-0.34) | <0.001 |
| Renal agenesis and dysgenesis |  |  |
| No | Ref |  |
| Yes | - | 0.988 |
| Urinary tract infection |  |  |
| No | Ref |  |
| Yes | - | 0.983 |
| Sepsis |  |  |
| No | Ref |  |
| Yes | 3.09 (1.61-5.91) | <0.001 |
| Respiratory distress syndrome |  |  |
| No | Ref |  |
| Yes | 2.50 (1.59-3.95) | <0.001 |
| Asphyxia |  |  |
| No | Ref |  |
| Yes | - | 0.984 |
| Patent ductus arteriosus |  |  |
| No | Ref |  |
| Yes | 7.63 (4.98-11.71) | <0.001 |
| Necrotizing enterocolitis |  |  |
| No | Ref |  |
| Yes | 1.89 (0.66-5.43) | 0.235 |
| Heart rate | 1.02 (1.01-1.04) | 0.001 |
| Respiratory rate | 0.99 (0.97-1.01) | 0.273 |
| Bicarbonate | 0.97 (0.89-1.04) | 0.367 |
| Sodium | 1.05 (1.00-1.10) | 0.062 |
| Potassium | 0.83 (0.66-1.04) | 0.100 |
| Chloride | 1.10 (1.05-1.15) | <0.001 |
| Urine output | 1.01 (1.01-1.01) | <0.001 |
| Mechanical ventilation |  |  |
| No | Ref |  |
| Yes | 3.80 (2.01-7.19) | <0.001 |
| Vasopressor |  |  |
| No | Ref |  |
| Yes | 6.41 (4.08-10.08) | <0.001 |
| Vancomycin |  |  |
| No | Ref |  |
| Yes | 4.47 (2.88-6.94) | <0.001 |
| ACE inhibitor |  |  |
| No | Ref |  |
| Yes | 8.67 (0.78-96.42) | 0.079 |
| Nonsteroidal anti-inflammatory drug |  |  |
| No | Ref |  |
| Yes | 6.55 (4.26-10.06) | <0.001 |
| Amphotericin B |  |  |
| No | Ref |  |
| Yes | - | 0.986 |
| Acyclovir or valacyclovir |  |  |
| No | Ref |  |
| Yes | 6.92 (2.41-19.83) | <0.001 |

AKI=acute kidney injury; ACE=angiotensin-converting enzyme; OR=odd ratio; CI=confidence interval.
