# Supplementary material for: Total bilirubin level is associated with acute kidney injury in neonates admitted to the neonatal intensive care units: based on MIMIC-III database
Source: Eur J Pediatr. 2024 Jul 11;183(10):4235–41. doi: 10.1007/s00431-024-05682-5 (PMC11413182; doi:10.1007/s00431-024-05682-5)
Supplement: Supplementary file 3 — Supplementary file3 (DOCX 15 KB) [file 431_2024_5682_MOESM3_ESM.docx]

Supplemental Table 3 Collinearity test by variance inflation factor

| Variables | VIF |
| --- | --- |
| Birth weight | 1.66852 |
| Sepsis | 1.22472 |
| Respiratory distress syndrome | 1.93835 |
| Patent ductus arteriosus | 1.65370 |
| Heart rate | 1.08225 |
| Chloride | 1.16495 |
| Urine output | 1.39888 |
| Ventilation | 1.80008 |
| Vasopressor | 1.28096 |
| Vancomycin | 1.63600 |
| Nonsteroidal anti-inflammatory drug | 1.98735 |
| Acyclovir or valacyclovir | 1.03213 |
